# Primary Synovial Chondromatosis Displacing Popliteal Neurovascular Structures: A Case Report

**DOI:** 10.7759/cureus.93144

**Published:** 2025-09-24

**Authors:** Ayla M Osborn, William C Bogar, Yuri Korvatko, Hector Rivera-Melo

**Affiliations:** 1 Diagnostic Imaging, National University of Health Sciences, Lombard, USA

**Keywords:** benign neoplasms, imaging modalities, knee joint pain, neurovascular complications, synovial chondromatosis

## Abstract

The purpose of this case report is to raise awareness among clinicians about primary synovial chondromatosis (PSC), including its pathology, imaging findings, and potential complications. Our patient experienced a slip and fall during icy conditions, prompting radiographs that revealed an incidental left knee lesion. A biopsy confirmed synovial chondromatosis, but the patient did not seek treatment at that time. Months later, he noticed a decrease in his range of motion, along with discomfort and swelling in the left knee. Over one year after the fall, the patient presented to our facility with a newly developing limp. Radiographs and diagnostic ultrasound of the left knee revealed a moderate-sized knee joint effusion and numerous small calcific densities of similar size, located both anterior and posterior to the knee joint. There was also slight internal color Doppler flow within the knee lesion. Follow-up MRI with and without intravenous contrast further showed contrast enhancement and significant mass effect with displacement of the popliteal neurovascular bundle. Due to the complex nature of this lesion, the patient was evaluated by an orthopedic oncologist and treated with a complex limb salvage procedure performed in conjunction with a vascular surgeon. Patients with knee pain and imaging findings suggestive of PSC warrant further investigation due to the potential for progression and adverse outcomes when diagnosis or treatment is delayed.

## Introduction

Primary synovial chondromatosis (PSC) is a rare benign neoplasm that develops in approximately 1.8 cases per million per year [1]. The pathology is characterized by the production of hyaline cartilage nodules, which may detach and deposit in a joint, bursa, or tendon sheath [2,3]. The disease process can be asymptomatic but often manifests with insidious onset of local pain and swelling with decreased range of joint motion. Additionally, joint tenderness and crepitus may be found on physical examination. Here, we present an unusual case of PSC of the knee with significant local displacement of the popliteal structures. Based on our literature search, this degree of local encroachment on the popliteal neurovascular bundle in PSC has not been frequently reported. A further complication of malignant transformation to chondrosarcoma has been reported in 5-10% of cases of PSC [1]. The goal is to raise awareness among clinicians to include PSC in the differential diagnosis when evaluating knee complaints and to foster timely clinical management in such cases.

## Case presentation

**Figure 1:**
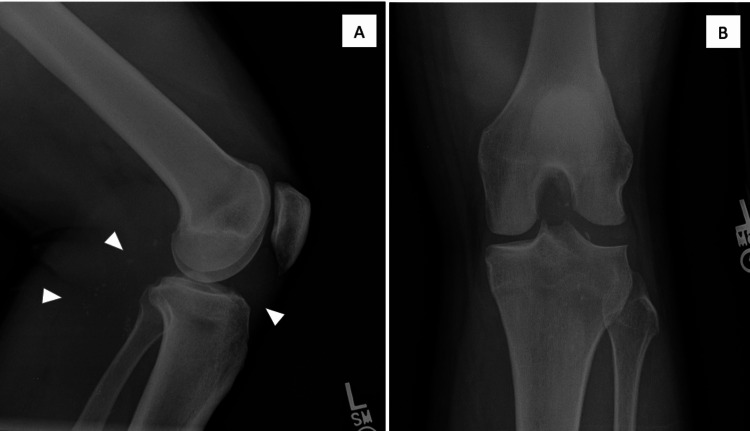
(A) Lateral and (B) tunnel radiographic views of the left knee showing multiple loose bodies. Radiographic examination revealed multiple small intraarticular loose bodies, both posterior and anterior to the joint line (arrowheads), extending into the popliteal space and posterior upper leg.

A 55-year-old male presented to our clinic with a newly developing limp, worsening left ankle swelling, and left calf pain. The patient’s medical history included bronchial asthma, Barrett esophagus, hypothyroidism, and seasonal allergies. Additional clinical information included a biopsy-proven diagnosis of PSC of the left knee from approximately one year prior. Following the diagnosis, the patient experienced continued swelling and a growing mass with decreased range of motion in his left knee. Prolonged sitting reportedly aggravated the patient’s symptoms. Left knee radiographs and diagnostic ultrasound obtained at our facility revealed a popliteal mass with multiple similarly sized small calcific nodules and a complex joint effusion (Figure 1A-1B, 2A-2C). Subsequent contrast-enhanced MRI of the left knee identified an infiltrative, lobulated, and irregularly enhancing mass with significant invasion of the anterior and posterior knee compartments (Figure 3A-3D). Following clinical and radiological assessment, the patient was referred to an orthopedic oncologist and ultimately underwent resection of the posterior mass. A complex limb salvage procedure with neurovascular dissection was required due to the lesion’s close proximity to the neurovascular bundle. The surgical report indicated that a synovectomy was not performed. A 15.0 x 7.5 x 5.5 cm mass was excised, and pathology confirmed the diagnosis of synovial chondromatosis (Figure 4). Following postoperative recovery, the patient reported improvement and reduction in his symptoms of ankle and knee swelling and pain.

**Figure 2:**
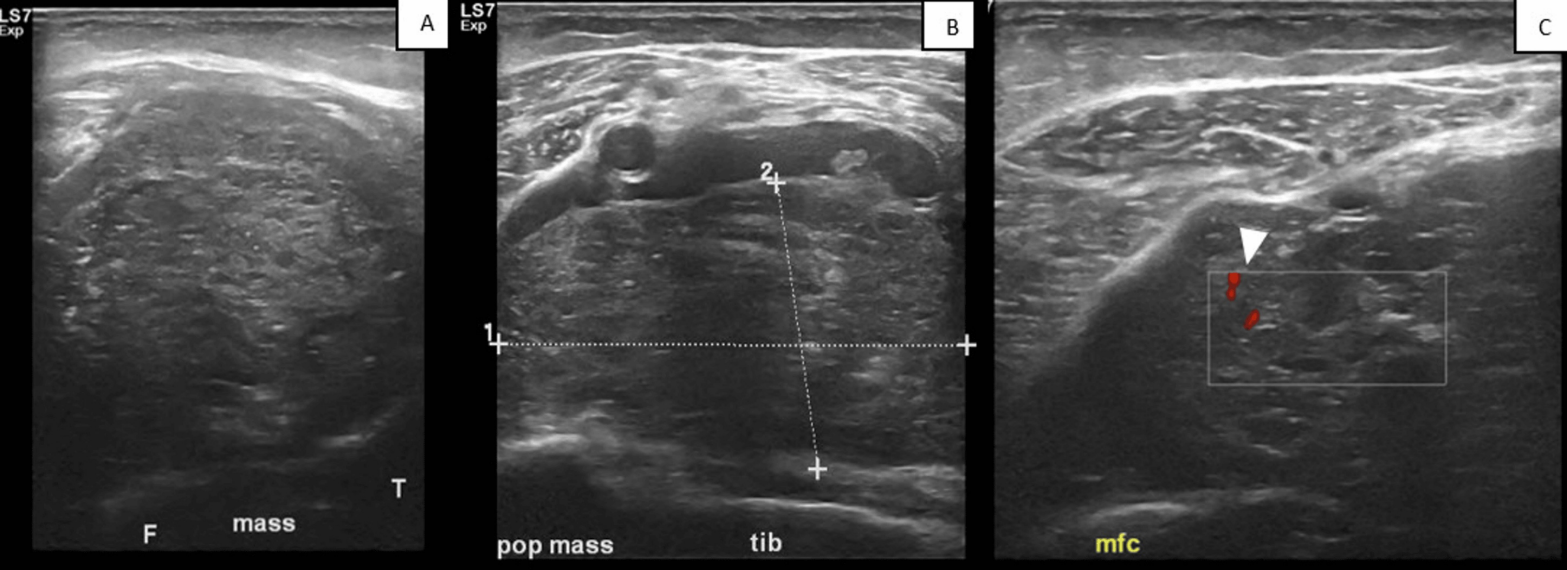
(A-C) Transverse ultrasound images of the left popliteal region. Transverse ultrasound images (A-C) showing a large heterogeneous soft tissue mass with multiple calcified fragments and slight internal color Doppler activity (arrowhead). The mass extensively involves the popliteal space, potentially affecting regional neurovascular structures. Reported measurements are approximately 5-6 cm (sagittal) x 4-6 cm (anteroposterior) x 4-6 cm (transverse).

**Figure 3:**
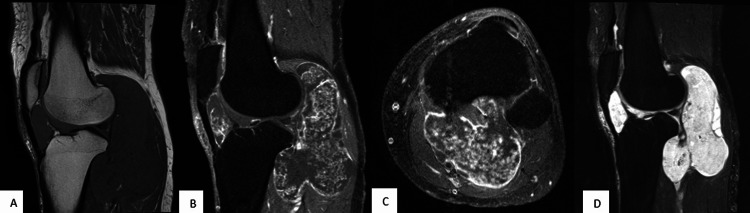
MRI of the left knee showing a mass located anterior and posterior to the joint. MRI, including sagittal T1W (A), sagittal T1W+C fat suppressed (B), axial T1W+C fat suppressed (C), and sagittal T2W fat suppressed (D), reveals a large lobulated lesion with components both anterior and posterior to the knee joint. There is irregular internal and rim-like outer enhancement. These findings additionally confirm the extensive and invasive nature of the lesion with significant displacement of posterior knee structures, including the popliteal neurovascular bundle. MR reported measurements: 13.4 cm craniocaudal x 8.1 cm x 5.9 cm (maximal transaxial dimension).

**Figure 4:**
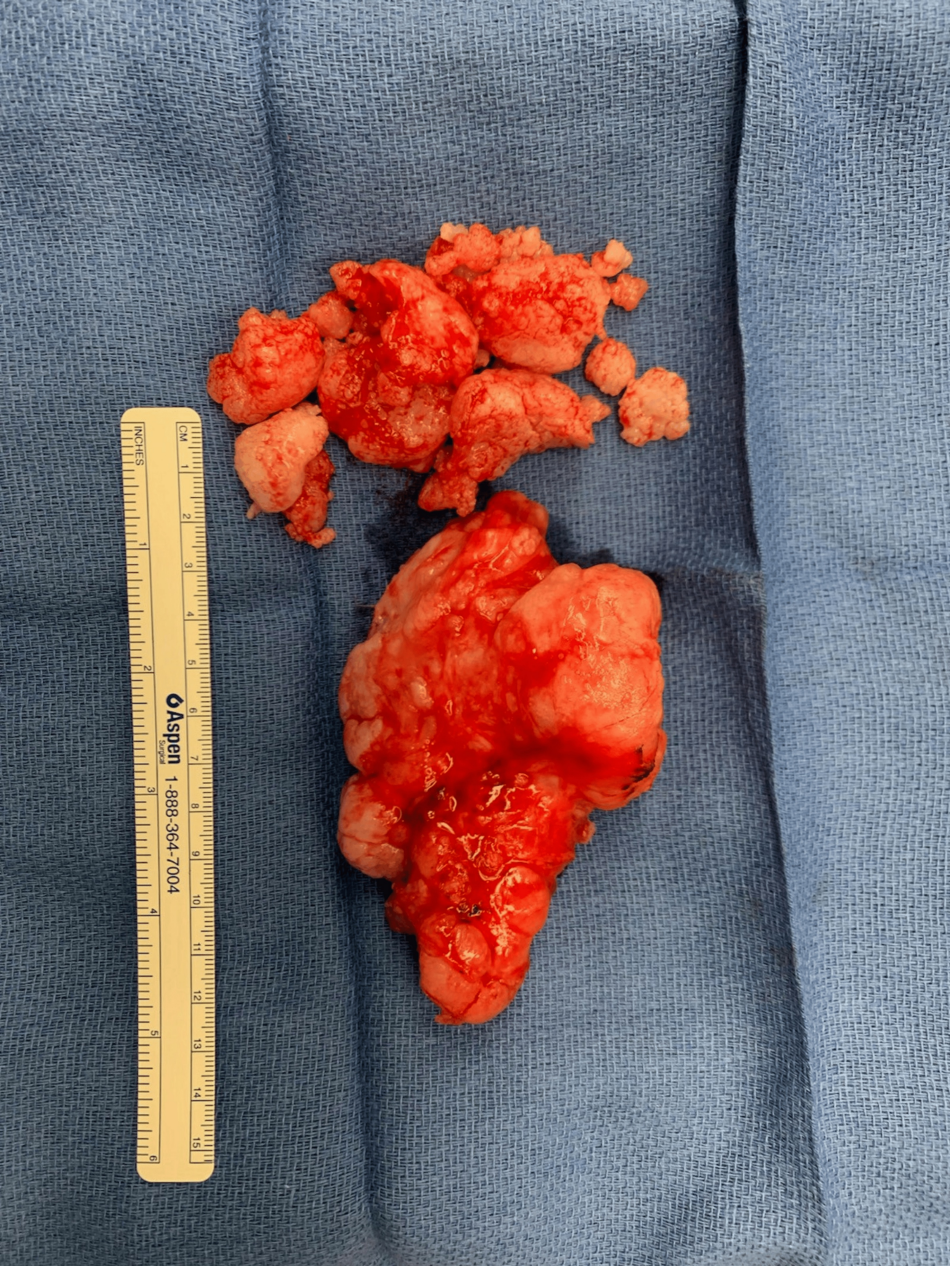
Surgical specimen. The excised lesion is described by pathology as an un-oriented, white-tan, lobulated, fragile mass (15.0 x 7.5 x 5.5 cm). No discoloration or necrosis was observed.

## Discussion

PSC is a rare benign neoplasm of uncertain etiology, which may progress or sometimes be self-limiting [2,4]. The condition may be asymptomatic, but the clinical picture often involves insidious onset of joint pain, swelling, and decreased range of motion that coincides with joint tenderness, crepitus, and palpable nodules on physical examination [1,4]. Due to the insidious nature of this pathology, diagnosis is often delayed by an average of five years, allowing for subsequent complications and a less favorable prognosis [2]. Therefore, PSC should be considered in the differential diagnosis when assessing joint concerns.

Males are affected two to four times as often as females, with the typical age of onset in the third through fifth decades of life. PSC most commonly involves the knee, but any synovial joint can be affected [3]. PSC should be differentiated from secondary synovial chondromatosis, which develops as a result of degenerative joint disease, trauma, or other joint pathology leading to accumulation of intraarticular calcific or osteocartilaginous loose bodies.

Despite its benign histological origin, PSC may lead to significant regional complications, including progressive damage to articular cartilage and adjacent bone, development of premature osteoarthritis, and rarely, malignant transformation to chondrosarcoma [2]. This case highlights the potential for the lesions to be locally disruptive and the importance of early and correct diagnosis to limit further complications. The optimal treatment of PSC consists of surgical resection of intraarticular loose bodies and sometimes synovectomy to prevent recurrence and other potential complications [1]. Recurrence rates are reported between 3% and 23% [2]. To our knowledge, very few cases of this condition with locally disruptive features of this nature have been reported in the knee. One case report described complications in a hip with mass effect on the femoral artery, and another reported venous obstruction leading to venous stasis ulcers [5,6].

Radiological evaluation of PSC plays a significant role in its diagnosis. Typical radiographic findings of PSC include multiple intraarticular loose bodies of uniform size with varying amounts of mineralization [7]. Occasionally, extrinsic pressure erosion of adjacent cartilage or bone has been reported [2,8]. Radiographs may be normal in up to 30% of cases; therefore, MRI may play a crucial role in the diagnosis and identification of regional complications. MRI commonly demonstrates a lobulated mass with predominantly low to intermediate signal on T1W and high signal on T2W sequences, with low-intensity foci on all pulse sequences due to mineralized or ossified components [2,4]. Ultrasound demonstrates a heterogeneous mass containing numerous hyperechoic foci with related acoustic shadowing, confirming the presence of intraarticular calcified fragments. Typically, PSC lesions do not demonstrate internal color or power Doppler flow on ultrasound [2]. An advantage of ultrasound is its relatively low cost and ease of access compared to other modalities. This provides clinicians and patients with an alternative imaging modality for the characterization of joint findings.

As demonstrated in our case, both radiographic and sonographic examinations may reveal numerous intraarticular calcific foci and joint effusion. Ultrasound was able to demonstrate significant extension of this mass into the popliteal space and the posterior upper leg. In addition, sonographic findings of abnormal internal color Doppler flow within the mass raised concerns about potential regional complications. Increased clinical awareness of this pathology and knowledge of its early radiological assessment allow for accurate diagnosis, patient education, and treatment, offering a potentially more favorable outcome.

## Conclusions

This case provides a unique learning opportunity for clinicians to develop a high level of awareness of PSC and its potential complications. Including this pathology in the differential diagnosis of joint complaints may allow timely detection of such lesions and significantly improve the outcome by preventing potential complications.

## References

[1] Memon AH, Din NU (2025). Memon AH, Din NU. Synovial & tenosynovial chondromatosis. PathologyOutlines.com. https://www.pathologyoutlines.com/topic/jointssynovialosteochondromatosis.html.

[2] Murphey MD, Vidal JA, Fanburg-Smith JC, Gajewski DA (2007). Imaging of synovial chondromatosis with radiologic-pathologic correlation. Radiographics.

[3] Resnick D, Jacobson JA, Chung CB, Kransdorf MJ, Pathria M (2024). Resnick’s Bone and Joint Imaging Fourth Edition. https://www.inspectioncopy.elsevier.com/book/details/9780323523271.

[4] Pal SK, Srikant K, Dash A (2021). A case of synovial chondromatosis of knee joint treated with total knee arthroplasty. J Orthop Trauma Rehabil.

[5] van der Valk MR, Veltman ES, Assink J, Veen MR (2019). Synovial chondromatosis of the hip, a case report and literature review. J Orthop.

[6] Herrero C, Lemmens C, Sloves J, Tejwani N, Maldonado TS (2018). Synovial chondromatosis in patient presenting with chronic venous stasis ulcers. Ann Vasc Surg.

[7] Maghear L, Serban O, Papp I, Otel O, Manole S, Botan E, Fodor D (2018). Multimodal ultrasonographic evaluation in a case with unossified primary synovial osteochondromatosis. Med Ultrason.

[8] Serbest S, Tiftikçi U, Karaaslan F, Tosun HB, Sevinç HF, Balci M (2015). A neglected case of giant synovial chondromatosis in knee joint. Pan Afr Med J.

